# Bridging the Survival Gap: Strengthening Post-Discharge Care for Preterm Infants in Tanzania

**DOI:** 10.5334/aogh.5442

**Published:** 2026-08-25

**Authors:** Erick Mahatara, Kelvin Musa, Beatrice Jerome, Linda Paul Athman

**Affiliations:** 1 School of Medicine, KCMC University, Moshi, Tanzania; 2 Department of Paediatrics, Kilimanjaro Christian Medical Center (KCMC), Moshi, Tanzania; 3 Department of Paediatrics and Child Health, Muhimbili University of Health and Allied Sciences, Dar-es-Salaam, Tanzania

**Keywords:** neonatal mortality, post-discharge care, Tanzania, mHealth, NEST360, ECEM-TZ, health financing

## Abstract

*Background:* Despite notable progress in inpatient neonatal care expansion in Tanzania, expanding from 14 facilities in 2018 to 362 by 2025, a critical survival gap persists during the post-discharge transition. National policies heavily prioritize pre-discharge mortality, leaving the high-risk, 60-day post-discharge window unaddressed. This study evaluates targeted policy, financing, and digital health interventions designed to mitigate post-discharge neonatal mortality.

*Methods:* We analyzed integrated implementation pathways within the Tanzanian health sector, specifically examining the institutional feasibility, scaling capacity, and clinical outcomes of the NEST360 Implementation Tracker (NEST-IT) and the Essential Coaching for Every Mother Tanzania (ECEM-TZ) mobile health framework.

*Results:* Implementation data demonstrate that NEST-IT is a highly viable data-surveillance tool, achieving a 93% feasibility rate for daily clinical use and providing real-time data to drive national quality improvement targets. Concurrently, the ECEM-TZ digital platform effectively circumvents nurse-midwife resource limitations by providing a 54-message educational framework that improves caregiver knowledge and triples postnatal check-up attendance.

*Conclusion:* Eliminating post-discharge mortality requires expanding longitudinal care frameworks. To achieve Tanzania’s national target of 15 neonatal deaths per 1000 live births, strategic imperatives must include institutionalizing zero-rated mHealth tools, formalizing community health worker follow-up protocols, and explicitly integrating specialized outpatient neonatal services into the standard benefit package of the 2026 universal health insurance rollout.

## Background

The landscape of neonatal health in the United Republic of Tanzania has undergone a profound transformation, characterized by substantial progress in reducing under-five mortality alongside the persistent challenge of neonatal deaths. An estimated 2.3 million neonatal deaths occur annually worldwide [1]. While the country has successfully reduced maternal and child mortality by approximately 60% over the past 20 years, the decline in neonatal mortality has remained comparatively slow, currently estimated at 24 deaths per 1000 live births according to the 2022/2023 Tanzania Demographic and Health Survey (TDHS) [1]. Prematurity remains a major contributor to neonatal mortality in Tanzania. Previous national estimates attributed approximately 27.6% of neonatal deaths to preterm birth, making it one of the leading causes of neonatal mortality [2].

Prematurity acts as a primary determinant of infant survival and quality of life, with the highest burden, approximately 95.6%, concentrated in low-income settings, where preterm birth rates are rising [3]. While interventions such as establishing specialized Neonatal Intensive Care Units (NICUs) and scaling up Kangaroo Mother Care (KMC) have improved the initial survival of preterm infants within hospital walls, systemic challenges persist. These include limited availability of adequately equipped units, a shortage of trained neonatal healthcare providers, and high out-of-pocket costs for prolonged care [4]. Consequently, many infants are discharged early, often at weights between 1.5 kg and 1.8 kg, before achieving full physiological maturity. This creates a critical clinical frontier in which post-discharge survival depends heavily on caregiver capacity, home environment, and continuity of care, areas that remain insufficiently addressed in national post-discharge systems.

Despite increased facility-based survival of preterm infants, post-discharge mortality and morbidity remain high. Approximately 3.1% of neonates discharged from neonatal wards die within 60 days, with 26.8% of these deaths occurring within the first week following discharge [5]. This survival gap is driven by inadequate maternal knowledge of essential newborn care, poor adherence to recommended feeding practices like exclusive breastfeeding, and exposure to infections and poor adherence to thermal care [6]. Furthermore, the weak linkage between facility care and community-based follow-up systems prevents the timely identification of deteriorating infants, leading to high readmission rates and community-based fatalities; for instance, the mortality rate for readmitted neonates at Muhimbili National Hospital was recorded at 4.8% [7].

## Problems or Challenges

1. **Health system constraints**
  The Tanzanian health system faces significant structural barriers in providing standardized neonatal follow-up. While national guidelines for the establishment of Neonatal Care Units (NCUs) were launched in 2019, implementation has been uneven. A multi-region survey revealed that while 48% of facilities had established some form of NCU, only a negligible 3% met the minimum national standards for a fully functional unit, which requires a NICU/HDU, a KMC ward, and a general neonatal ward [8]. This lack of infrastructure is coupled with a “know-do gap” in which adherence to core guidelines for thermal care and sepsis management often falls short of the WHO-recommended 80% standard [9].
2. **Socioeconomic barriers**
  The economic burden of preterm care is a primary driver of the survival gap. For many families, the cost of prolonged neonatal intensive care is catastrophic; in one documented case, the hospital bill for a preterm infant reached 50 million Tanzanian Shillings after three months of care [10]. While the National Health Insurance Fund (NHIF) provides a benefit package covering registration, consultation, and oxygen therapy as of March 2024, many medical consumables and medications not on the National Essential Medicines List (NEMLIT) require out-of-pocket payments [11]. Geographic barriers further exacerbate this issue, as rural families often live over 100 km away from specialized facilities, making follow-up visits a difficult journey through rough terrain that consumes household resources and time [12].
3. **Maternal and caregiver knowledge gaps**
  Post-discharge care often depends on caregivers recognizing danger signs and sustaining feeding, thermal care, and follow-up practices, yet significant knowledge gaps remain. A study in Central Tanzania found that only 38% of postnatal mothers had adequate knowledge about KMC, with factors such as younger age and lower education levels predicting poorer outcomes [13]. Many mothers struggle with the sustainable practice of KMC at home, often failing to achieve the recommended daily dose/duration of 20 or more hours due to competing domestic responsibilities like fetching water and cooking. Adolescent mothers are particularly vulnerable, frequently lacking the confidence to bathe a fragile preterm infant and succumbing to cultural pressures to introduce solids early when they perceive their breast milk supply to be insufficient [14].
4. **Environmental and infection risks**
  The home environment often presents substantial risks to fragile preterm infants. Overcrowding and suboptimal sanitation in many households increase susceptibility to infectious diseases, with presumed sepsis and pneumonia accounting for over 70% of post-discharge deaths. Infants discharged at low weights are particularly vulnerable, with mortality rates as high as 20.6% in a case study in Uganda, primarily due to sepsis and suspected cot death [15]. These risks are compounded by limited access to improved drinking water; research indicates that households using improved water sources have lower odds of under-five mortality, highlighting the impact of the environmental outer setting on newborn survival [16].

## Proposed Key Pathways/Interventions

### Strengthening community-based follow-up systems

To reach families in remote areas, Tanzania must integrate community health workers (CHWs) into formal neonatal follow-up protocols. Integrated home-visiting interventions that focus on health, nutrition, and responsive stimulation have been shown to benefit child cognitive development and linear growth in rural Tanzania. Large-scale CHW interventions in Dar es Salaam have successfully identified pregnant women early in their homes and nearly halved the probability of women delivering at home (3.9% vs 7.3%) [17]. By conducting scheduled post-discharge home visits, CHWs can serve as a structural support for families, providing normative social influence and identifying danger signs early, using digital apps to guide their work and improve reporting.

1. **Structured** **maternal** **education** **programs**
  Strengthening discharge preparation is essential for a smooth transition home. Standardized education packages, such as the Essential Coaching for Every Mother Tanzania (ECEM-TZ) program, can strengthen discharge preparation by providing structured counseling and continued postpartum support. By utilizing mobile technology to provide 54 unique text messages from birth to six weeks postpartum, the program addresses critical gaps in breastfeeding self-efficacy and the recognition of neonatal danger signs, such as fever and respiratory distress [18]. To achieve national scale, this intervention must be institutionalized within the Ministry of Health’s national mHealth architecture and integrated into the 2026 rollout of universal health insurance as a reimbursable preventive service [19]. Furthermore, scaling requires strategic public–private partnerships with telecommunication providers to “zero-rate” these messages, ensuring that financial barriers do not prevent the most vulnerable mothers from accessing life-saving digital education. Supplementing in-person counseling with such standardized, zero-rated digital tools not only improves maternal self-efficacy but also mitigates the systemic challenges faced by nurse-midwives who lack the time for comprehensive discharge preparation [20].
2. **Scaling up kangaroo mother care beyond facilities**
  KMC must move beyond hospital wards to become a community-supported practice. Evidence has estimated that community-initiated and immediate KMC can reduce neonatal deaths by 25% compared to waiting for infant stabilization [21]. Scaling this intervention requires training families on safe skin-to-skin practices and establishing community-based KMC support groups to address sociocultural attitudes. Innovative “Zero Separation” units, such as the one launched in Kwimba District, provide a model for involving both mothers and fathers in care, which helps stabilize premature babies weighing less than 1.5 kg and humanizes the transition to home [12].
3. **Improving nutritional support**
  Promotion of exclusive breastfeeding is the primary nutritional intervention for preventing post-discharge mortality. Because growth failure is prevalent among preterm survivors, with 38.7% of infants in some cohorts exhibiting growth failure by 12 weeks post-discharge, nutritional counseling for lactating mothers must be prioritized [22]. This includes teaching mothers to feed at least eight times per day and managing the “perceived insufficiency” of milk through professional and peer support [23]. In vulnerable populations, mobile-based reminders for exclusive breastfeeding and remote support have been shown to reduce the risk of cessation by 25% [24].
4. **Policy and health financing interventions**
  Socioeconomic strain contributes significantly to post-discharge mortality. Recent evidence suggests that high-risk infants in sub-Saharan Africa face death in the first 60 days following hospital release, yet national policies often prioritize pre-discharge outcomes over longitudinal monitoring [25]. To mitigate this, integrated health financing must transition toward supporting community-based follow-up and community KMC through its trained health workers [26]. Furthermore, the 2026 rollout of universal health insurance presents a pivotal opportunity to include specialized outpatient neonatal services within the standard benefit package, ensuring that the continuum of care extends beyond the hospital walls to prevent late-onset complications and financial catastrophe for vulnerable families [19].
5. **Strengthening data and surveillance**
  High-quality data must drive effective policy. Currently, Tanzania’s national health information systems often lack comprehensive tracking for post-discharge outcomes and gestational age, making it difficult to define prematurity-specific mortality trends [1]. Strengthening the Maternal and Perinatal Death Surveillance and Response (MPDSR) system is essential for moving from data to action [1]. The NEST360 Implementation Tracker (NEST-IT) has proven to be a transformative tool in this regard, demonstrating a 93% feasibility rate for routine clinical use and ensuring 100% engagement in data-driven quality improvement projects across implementing sites [27]. By providing real-time feedback loops and identifying documentation gaps, NEST-IT allows facility teams to set precise targets, such as the national target of 15 neonatal deaths per 1000 live births. Scaling this intervention to all regions is a viable and strategic priority for achieving sustained neonatal survival [28, 29].

## Expected Outcomes

The implementation of these integrated pathways is expected to improve survival rates among preterm infants post-discharge. Facilities implementing the NEST360 bundle, which includes clinical mentoring and qualified technologies, have already shown statistically significant relative reductions of 14–54% in adjusted inpatient neonatal mortality. Increased maternal competence in providing specialized care, such as continuous KMC and early recognition of danger signs through programs like ECEM-TZ, will empower families and reduce the reliance on overstretched tertiary facilities [18].

Furthermore, these interventions are expected to reduce readmissions due to preventable complications like sepsis and hypothermia, which are currently major drivers of post-discharge fatalities. Ultimately, a strengthened continuum of care supported by expanded NHIF coverage and a larger network of 362 hospitals will ensure that preterm infants not only survive the neonatal period but could also achieve better long-term growth and neurodevelopmental outcomes [30]. This holistic approach will foster a more resilient health system capable of protecting its most vulnerable citizens.

## Conclusion

Reducing mortality from prematurity in Tanzania requires a paradigm shift from a facility-centric approach to a continuum-of-care model that extends into households and communities. While hospital-based innovations like the NICU and KMC have saved many lives, the “survival gap” after discharge remains a lethal hurdle for the country’s most fragile infants. Currently, the Ministry of Health and TAMISEMI have adopted the “Begin with What You Have” strategy to maximize existing resources, namely NICUs, and have successfully decentralized premature care to 362 facilities [8, 30]. However, setbacks persist, including the fact that only 3% of facilities meet the full NCU standard, and high out-of-pocket costs remain a barrier for the most vulnerable.

To close this gap, Tanzania must strengthen approaches that link facility-based neonatal care with community-based follow-up. This should include the formal integration of CHW home visiting protocols into national policy to identify post-discharge danger signs and provide emotional support. The government should prioritize the national scale-up of mHealth tools like ECEM-TZ to bridge the knowledge gap for mothers, particularly adolescents and rural caregivers who face the highest risk. Furthermore, the rollout of Universal Health Insurance in 2026 must explicitly include comprehensive subsidies for neonatal intensive care and specialized follow-up products to eliminate financial toxicity. By leveraging these technologies, empowering community cadres, and ensuring equitable financing, Tanzania can ensure that preterm infants are allowed to not only survive but thrive throughout their life course.

## Data Availability

Data and materials have been obtained from online, authorized published documents, reports, and articles.
